# Gram-Negative Anaerobic Spondylodiscitis Caused by Prevotella intermedia: A Case Report

**DOI:** 10.7759/cureus.90551

**Published:** 2025-08-20

**Authors:** Chih-Yung Chiang, Kai-Chiang Yang, Feng-Huei Lin, Chang-Chin Wu

**Affiliations:** 1 Department of Biomedical Engineering, National Taiwan University, Taipei, TWN; 2 Department of Orthopedics, En Chu Kong Hospital, New Taipei City, TWN; 3 School of Dental Technology, College of Oral Medicine, Taipei Medical University, Taipei, TWN

**Keywords:** cauda equine syndrome, metronidazole, prevotella intermedia, pyogenic vertebral osteomyelitis, spondylodiscitis

## Abstract

Oral bacteria, such as *Prevotella intermedia*, can be a rare cause of spondylodiscitis, especially in patients who have recently undergone an esophagogastroduodenoscopy (EGD). Gram-positive bacteria are typically the most common infecting organisms in spondylodiscitis cases. However, if empirical antibiotic treatment fails, it is crucial to perform a biopsy of the infected disc and vertebral body to culture and identify any unusual pathogens, such as anaerobic bacteria. We report the case of a 43-year-old man with L5/S1 spondylodiscitis attributed to *P. intermedia*. Cauda equina syndrome developed two days later after initiation of empirical treatment with intravenous oxacillin, which prompted emergent decompression surgery. After initial decompression and debridement surgery, intravenous oxacillin was continued due to the negative intraoperative culture result. However, pus discharge from the surgical wound was noted six days after the first operation, and the C-reactive protein level was elevated. Re-operation for debridement was done, and* P. intermedia* was isolated from intraoperative tissue culture. According to antibiotic susceptibility testing, antibiotics were adjusted to intravenous metronidazole (7.5 mg/kg every six hours), and the patient’s clinical symptoms and signs improved gradually. The patient had undergone an EGD four weeks prior, and the bacteremia could have originated from the breach of the mucocutaneous barrier that occurred during the EGD, allowing oral bacteria to access the bloodstream. This report highlights an unusual case of *P. intermedia*-associated spondylodiscitis and emphasizes the potential for rare pathogens to cause serious spinal infections. Additionally, it aims to draw attention to the possibility that procedures such as EGD might introduce oral bacteria into the bloodstream, leading to infections distant from the site of the procedure.

## Introduction

In pyogenic vertebral osteomyelitis, the most significant infecting organism is *Staphylococcus aureus*, responsible for over 50% of cases in the reported series. *Streptococcus* species account for 20%, while anaerobes contribute only 2-3% [1]. *Prevotella intermedia*, a gram-negative, obligate anaerobic, non-spore-forming pathogenic bacterium that is involved in periodontal infections, is a very rare cause of vertebral osteomyelitis. After reviewing the English-language literature, only three cases of vertebral osteomyelitis caused by *P. intermedia* were found [2-4]. A new case of vertebral osteomyelitis caused by *P. intermedia* is reported in this study.

## Case presentation

A 43-year-old man arrived at the emergency department (ED) with a chief complaint of severe low back pain that had persisted for two days. He reported that he had intermittent low back pain for years. However, the pain has become more severe in recent days, radiating along the L5/S1 dermatome to the bilateral posterior thigh and calf. He described the pain as a 9 out of 10 in intensity, sharp, constant, and worsened by movement. He reported no weakness in the limbs and denied any issues with fecal or urinary incontinence. The patient had been experiencing gastroesophageal reflux disease (GERD) for the last two months, and four weeks ago, an esophagogastroduodenoscopy (EGD) revealed a gastric ulcer, and he has since been on proton pump inhibitors (PPIs) for treatment.

The physical examination indicated that the patient's body temperature was 37.1°C, pulse rate was 90 beats per minute, blood pressure was 140/77 mmHg, and respiration rate was 19 breaths per minute. He had diffuse tenderness in his lower back, with the most pronounced sensitivity over the lower lumbar vertebrae. Laboratory tests indicated a significantly elevated white blood cell count of 15,900, with a remarkably high percentage of neutrophils, constituting 90.3% of the total. The C-reactive protein (CRP) level was 8.1 mg/dL.

**Figure 1:**
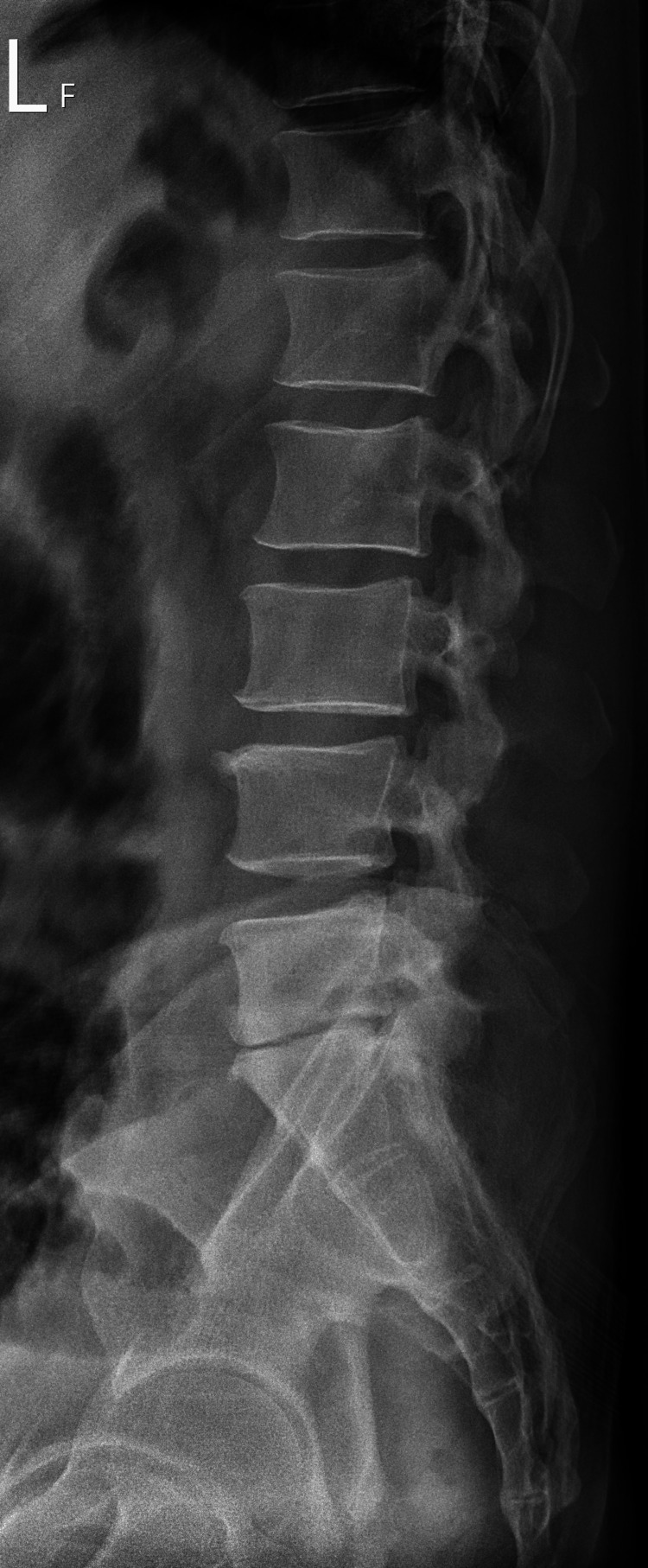
Radiography of the lumbar spine shows narrowing of the disc space at L5/S1 and some anterior marginal osteophytes.

**Figure 2:**
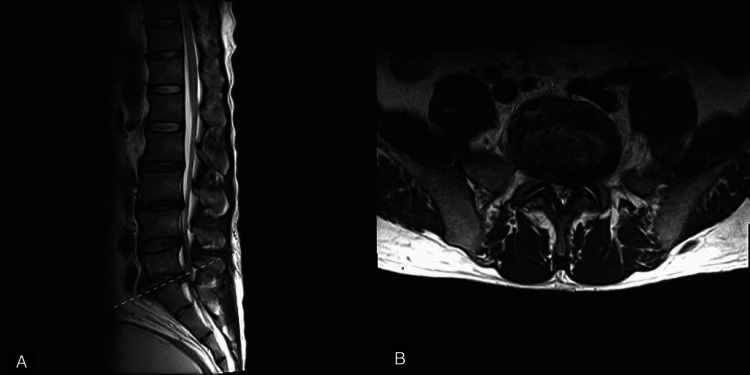
MRI of the lumbosacral spine. (A) The sagittal image shows irregular changes in the end plate with fluid accumulation at the L5/S1 disc space, suggestive of spondylodiscitis. Additionally, disc protrusions are noted at L3/4, L4/5, and L5/S1, along with spinal stenosis, which are most pronounced at L5/S1; (B) The axial view at the L5/S1 level (dashed line in A) reveals a disc protrusion with spinal stenosis, more prominent on the left side.

Radiography of the lumbar spine revealed a narrowing disc space at L5/S1 and some anterior marginal osteophytes (Figure 1). Magnetic resonance imaging (MRI) of the lumbosacral spine revealed irregular changes in the end-plate with fluid accumulation at the L5/S1 disc space, suggestive of spondylodiscitis (Figure 2). Additionally, disc protrusions were noted at L3/4, L4/5, and L5/S1, along with spinal stenosis, which was most pronounced at L5/S1.

The patient was empirically initiated on intravenous oxacillin at a dosage of 2 g every six hours, with pending blood cultures. However, following two days of antibiotic therapy, the patient developed an acute onset of weakness in the lower limbs, unsteady gait, sciatica, saddle anesthesia, and urinary retention. On physical examination, the patient showed paresis with 3/5 muscle strength in both lower limbs, along with absent ankle reflexes on both sides and a missing anal reflex. Given the acute onset of cauda equina syndrome, MRI was deferred, and emergent L5/S1 bilateral laminotomy for posterior decompression was conducted.

Frank pus was noted at the L5/S1 disc intra-operatively. However, two blood cultures taken from the patient in the ED and an intra-operative culture did not yield any pathogens, and oxacillin was kept. Pus discharge from the surgical wound was noted on day 6 postoperatively, and CRP was 10.3 mg/dL.

Debridement was performed, and both pus from the surgical wound and intraoperative biopsies culture yielded *P. intermedia*. Since *Prevotella* species are a common cause of periodontitis, the patient’s oral and periodontal condition was evaluated, and periodontitis was confirmed. Antibiotics were adjusted to intravenous metronidazole (7.5 mg/kg). The low back pain and cauda equina syndrome improved gradually. The CRP level was 1.0 mg/dL, and the patient was discharged after six weeks of intravenous metronidazole treatment. There was only mild numbness over his left posterior calf and mild difficulty in urination at the six-month follow-up.

## Discussion

Anaerobic gram-negative bacilli rarely cause spondylodiskitis or vertebral osteomyelitis; among these, *Prevotella* is an exceptionally rare infectious agent. Most studies investigating osteomyelitis of anaerobic bacterial origin point to *Clostridium* spp., *Fusobacterium* spp., and *Bacteroides* spp. as the typical causative agents. Searching the PubMed database using the keywords ‘*Prevotella* and spondylodiscitis’, ‘*Prevotella* and vertebral’, and ‘*Prevotella* and osteomyelitis’ uncovered eight reported cases of vertebral osteomyelitis in humans caused by different *Prevotella* species. However, only three of these cases were attributed to *P. intermedia* [2-4]. 

*P. intermedia *is commonly located in various parts of the human body, including the oral cavity, the digestive system, and the vaginal region. This bacterium frequently causes infections like periodontal diseases, infections of the pleura and lungs following aspiration, female genital infections, and abscesses within the abdomen. Nevertheless, it is usually considered a normal commensal rather than a pathogen when isolated in clinical specimens [5]. It is reasonable to deduce that spondylodiskitis in our patient was caused by *P. intermedia* because pus from a surgical wound and intraoperative biopsy culture both yielded *P. intermedia*. Also, the clinical symptoms and signs improved after antibiotics were adjusted to intravenous metronidazole, which is highly active against gram-negative anaerobic bacteria, such as *P. intermedia*.

In elderly individuals, vertebral osteomyelitis typically develops as a hematogenous infection [6]. Although previous research indicated that anaerobes comprised less than 1% of the organisms causing osteomyelitis, advancements in culture and isolation techniques have revealed an increasing incidence in more recent studies. Consequently, anaerobes are now more widely recognized in the pathology of osteomyelitis [2]. Our patient did not exhibit any traditional risk factors typically associated with the condition, such as a history of past back surgeries, diabetes mellitus, the presence of decubitus ulcers, a history of trauma, chronic sinusitis, or chronic renal failure [7]. One potential source of bacteremia in this patient might be the disruption of the mucocutaneous barrier during the EGD procedure. This disruption could have allowed the displacement of the oral flora into the bloodstream, resulting in bacteremia. Goyal et al. have documented a noteworthy case in which a patient developed vertebral osteomyelitis and epidural abscesses as a result of an infection with *Prevotella oralis*. This condition emerged after the patient underwent an EGD procedure [8].

In our patient, the identification of *P. intermedia* prompted further evaluation of the oral cavity, where periodontitis was confirmed. This finding highlights the potential role of oral infections as a reservoir for systemic dissemination of anaerobic bacteria. Periodontitis is a prevalent chronic condition, and *Prevotella* species are well-documented periodontal pathogens [5]. Previous studies have suggested that oral pathogens can enter the bloodstream through inflamed gingival tissue or following invasive procedures [9], thereby increasing the risk of extraoral infections, including spondylodiscitis. Therefore, in patients with spinal infections caused by anaerobic bacteria, a thorough oral examination should be considered to identify potential sources of bacteremia. This case underscores the importance of interdisciplinary collaboration between orthopedic surgeons, infectious disease specialists, and dental professionals in both diagnosis and management. Early recognition and treatment of periodontal disease may reduce the risk of systemic complications, such as vertebral osteomyelitis.

A review of the literature indicates that *P. intermedius* can lead to the formation of intravertebral abscesses, paravertebral abscesses, psoas abscesses, and epidural abscesses [4]. Vertebral destruction with spinal cord compression has also been reported [3]. In our case, cauda equina syndrome developed two days later after empirical therapy with intravenous oxacillin. It implied that *P. intermedialis* is not so kind, and the disease could progress quickly with inadequate treatment.

This case emphasizes the crucial need for an open surgical biopsy of the intervertebral disk space to both confirm the diagnosis and collect culture samples, particularly in the event that initial antimicrobial treatment aimed at gram-positive bacteria proves ineffective. It is essential that specimens from infected bones obtained during biopsy are routinely submitted for anaerobic cultures, given the increased frequency of anaerobic spondylodiskitis reports. Early administration of an appropriate antimicrobial agent is crucial in identifying an anaerobic pathogen. With timely diagnosis and appropriate treatment, the prognosis is generally favorable.

## Conclusions

In pyogenic vertebral osteomyelitis or spondylodiscitis cases, gram-positive bacteria are typically the most common infecting organisms. However, if empirical antibiotic treatment fails, it is crucial to perform a biopsy of the infected disc and vertebral body to culture and identify any unusual pathogens, such as anaerobic bacteria. If the patient underwent EGD in the previous four weeks, a possible source of bacteremia could be the breakage of the mucocutaneous barrier during the EGD.
